# Supplementary material for: Chromosome Rearrangement in Elymus dahuricus Revealed by ND-FISH and Oligo-FISH Painting
Source: Plants (Basel). 2023 Sep 14;12(18):3268. doi: 10.3390/plants12183268 (PMC10535892; doi:10.3390/plants12183268)
Supplement: Supplementary file 1 [file plants-12-03268-s001.zip › plants-2599005-supplementary.pdf]

Supplementary Materials

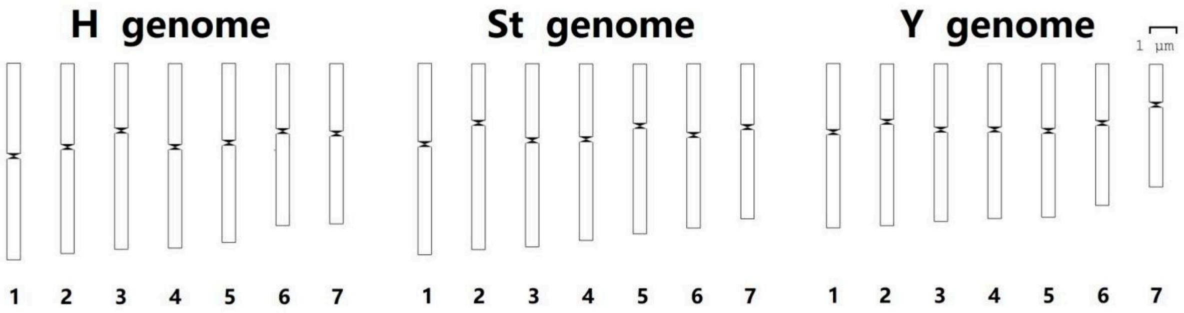

Figure S1. Ideogram for chromosomes of *E. dahuricus*. Within each genome, the chromosomes are arranged by the relative length. Bars, 1 μm.

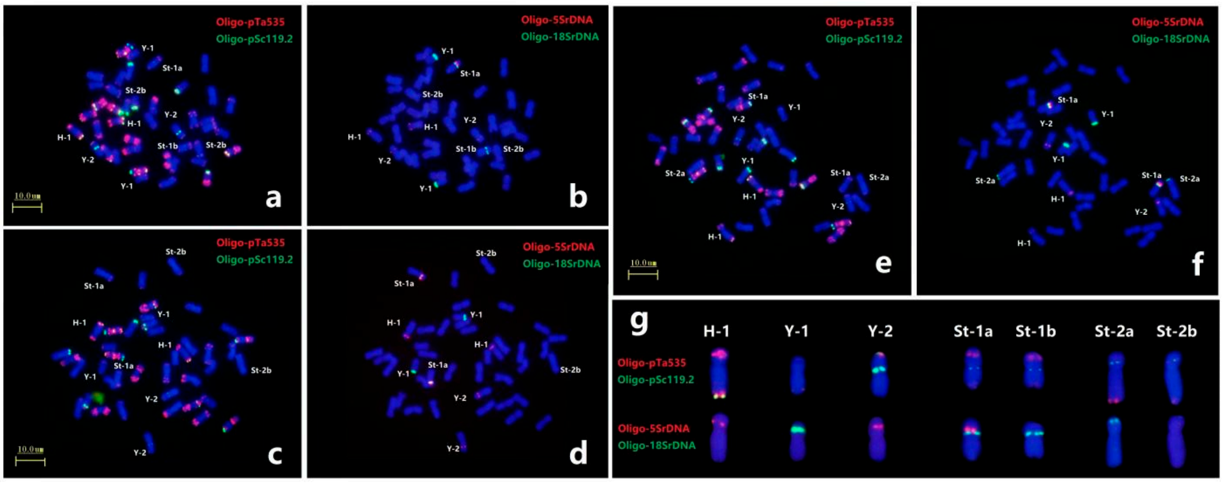

Figure S2. ND-FISH hybridization patterns showing the polymorphism of 18S and 5S rDNA sequences in *E. dahuricus*. The probes for FISH are: Oligo-pTa535 (red) + Oligo-pSc119.2 (green) (**a**, **c**, **e**); Oligo-18S rDNA +Oligo-5S rDNA (**b**, **d**, **f**). The karyotype of the H-1, Y-1, Y-2, St-1 and St-2 chromosomes are shown in(**g**).

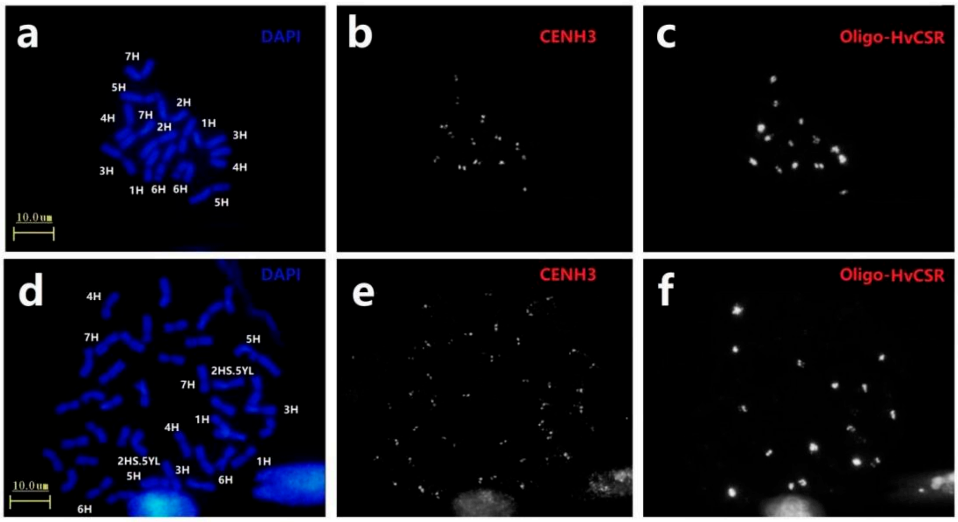

Figure S3. Comparative location of Oligo-HvCSR and anti-CENH3 in barley and *E. dahuricus*. DAPI-stained chromosomes are blue (**a**, **d**). Sequential immunostaining of anti-CENH3 (**b**, **e**) and ND-FISH of probe Oligo-HvCSR (**c**, **f**) in the same metaphase cells.

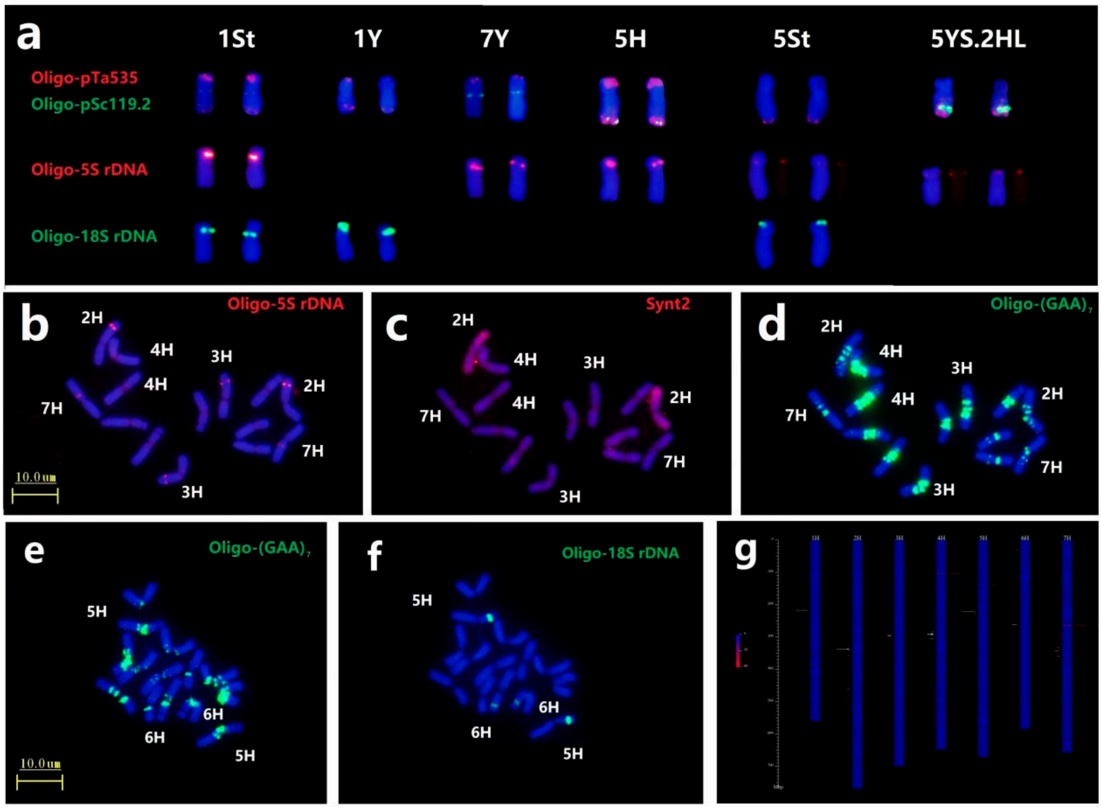

Figure S4. The karyotype of the 1St, 1Y, 7Y, 5H, 5St and 5YS.2HL chromosomes in *E. dahuricus* (a); Metaphase chromosomes in barley, the oligo probes are: Oligo-5S rDNA (**b**), Synt2 (**c**), Oligo-(GAA)$_7$ (**d, e**), Oligo-18S rDNA (f); visualized distribution of Oligo-5S rDNA in barley (*Hordeum vulgare* L.)-were indicated (g) by B2DSC web server.
